# Differential Chemical Components Analysis of *Periplocae Cortex*, *Lycii Cortex*, and *Acanthopanacis Cortex* Based on Mass Spectrometry Data and Chemometrics

**DOI:** 10.3390/molecules29163807

**Published:** 2024-08-11

**Authors:** Xianrui Wang, Jiating Zhang, Fangliang He, Wenguang Jing, Minghua Li, Xiaohan Guo, Xianlong Cheng, Feng Wei

**Affiliations:** 1 Institute for Control of Traditional Chinese Medicine and Ethnic Medicine, National Institutes for Food and Drug Control, Beijing 102629, China; niuyun006097@163.com (X.W.); 18341441178@163.com (J.Z.); hefangliang0602@126.com (F.H.); jingwenguang@nifdc.org.cn (W.J.); liminghua@nifdc.org.cn (M.L.); guoxiaohan@nifdc.org.cn (X.G.); 2 State Key Laboratory of Drug Regulatory Science, National Institutes for Food and Drug Control, Beijing 102629, China; 3 Institute for College of Traditional Chinese Medicine, China Pharmaceutical University, Nanjing 211198, China

**Keywords:** *Acanthopanacis Cortex*, *Lycii Cortex*, chemometric analysis, principal component analysis, *Periplocae Cortex*, partial least squares discriminant analysis

## Abstract

**Background:** *Periplocae Cortex* (PC), *Acanthopanacis Cortex* (AC), and *Lycii Cortex* (LC), as traditional Chinese medicines, are all dried root bark, presented in a roll, light and brittle, easy to break, have a fragrant scent, etc. Due to their similar appearances, it is tough to distinguish them, and they are often confused and adulterated in markets and clinical applications. To realize the identification and quality control of three herbs, in this paper, Ultra Performance Liquid Chromatography-Quadrupole Time of Flight Mass Spectrometry Expression (UHPLC-QTOF-MS^E^) combined with chemometric analysis was used to explore the different chemical compositions. **Methods:** LC, AC, and PC were analyzed by UHPLC-QTOF-MS^E^, and the quantized MS data combined with Principal Component Analysis (PCA) and Partial Least Squares Discriminant Analysis (PLS-DA) were used to explore the different chemical compositions with Variable Importance Projection (VIP) > 1.0. Further, the different chemical compositions were identified according to the chemical standard substances, related literature, and databases. **Results:** AC, PC, and LC can be obviously distinguished in PCA and PLS-DA analysis with the VIP of 2661 ions > 1.0. We preliminarily identified 17 differential chemical constituents in AC, PC, and LC with significant differences (*p <* 0.01) and VIP > 1.0; for example, Lycium B and Periploside H2 are LC and PC’s proprietary ingredients, respectively, and 2-Hydroxy-4-methoxybenzaldehyde, Periplocoside C, and 3,5-Di-O-caffeoylquinic acid are the shared components of the three herbs. **Conclusions:** UHPLC-QTOF-MS^E^ combined with chemometric analysis is conducive to exploring the differential chemical compositions of three herbs. Moreover, the proprietary ingredients, Lycium B (LC) and Periploside H2 (PC), are beneficial in strengthening the quality control of AC, PC, and LC. In addition, limits on the content of shared components can be set to enhance the quality control of LC, PC, and AC.

## 1. Introduction

The dried root bark of *Acanthopanax gracilistylus W. W. Smith*, a plant in the Araliaceae family, is known as *Acanthopanacis Cortex* (AC, Wu Jia Pi) [1,2]. It is believed to dispel wind and dampness, nourish the liver and kidneys, and strengthen tendons and bones [2]. Clinical practice treats rheumatic diseases, weak tendons and bones, physical weakness and fatigue, edema, and beriberi [3]. Recent research showed that as a potential new photosensitizer of head and neck squamous cell carcinoma, AC can upregulate the expression of Bax protein and Poly ADP-Ribose Polymerase-1-protein (PARP-1), further enhance the photodynamic therapy (PDT) effect, induce reactive oxygen species (ROS) production, and trigger the apoptosis pathway [4]. *Periplocae Cortex* (PC, Xiang Jia Pi), referring to the dried root bark of Periploca sepium Bge., has the effects of diuresis, reducing swelling, dispelling wind and dampness, and strengthening tendons and bones [2,5]. It is used clinically for lower limb edema, palpitations, shortness of breath, wind-cold-dampness bi syndrome, lumbosacral soreness, and weakness [5]. In addition, it has been proven that the chemical constituents contained in PC can effectively alleviate colonic inflammation, improve the intestinal epithelial barrier function, and prevent the occurrence of colitis and colitis-related tumors [6]. The *Lycii Cortex* (LC, Di Gu Pi) is the dried root bark of *Lycium Chinese* Mill of Solanaceae or *Lycium barbarum* L of Ningxia [7]. Its efficacy is mainly reflected in cooling the blood, removing steaming, clearing the lungs, and reducing internal heat. Therefore, it is commonly used in treating yin deficiency with tidal heat, bone steaming and night sweats, lung heat, cough, hemoptysis, nosebleeds, and internal heat and thirst [8,9]. LC, PC, and AC are all included in the 2020 edition of *Chinese Pharmacopoeia*. Their medicinal parts are all dried root bark, presented in a roll, single or double rolled, light and brittle, easy to break, and have a fragrant scent, and their cork layer has multiple rows of cells and contains starch granules. The three herbs have similar traits, making them readily confusing in markets and clinical applications. For example, many people mistake PC and LC for AC, and PC is utilized as AC or blended into AC for treatment. However, their clinical pharmacological actions differ from each other, and PC has toxicity. So, if the clinical application is inappropriate, it may directly threaten the patient’s life, resulting in irreparable damage. Therefore, to protect people’s lives, strengthen the market supervision and quality control of AC, PC, and LC, and avoid confusion about the use of the three herbs, it is essential to make use of modern scientific and technological methods to carry out the analysis of AC, PC, and LC.

To this end, numerous research studies have been conducted by various researchers from different perspectives. For example, using plant metabolomics and network pharmacology techniques, Li ZT et al. investigated and identified nine potential quality marker components of PC. One of these components is 4-methoxy benzaldehyde-2-O-β-d-xylopyranosyl-(1→6)-β-d-glucopyranoside, which can be used to differentiate PC harvested during the spring–autumn or summer seasons [10]. Zhang JX et al. established a rapid HPLC-ESI-MS method for the analysis of 24 components in LC, including 11 phenolic compounds, nine phenolic amides, and four cyclic peptides, of which cyclic peptides and phenolic amides were not only the abundant constituents but also the characteristic components for LC to be distinguished from the adulterants, and cyclic peptide was considered a chemical marker to distinguish LC from ones from different geographical regions [11]. Sun L et al. used an electronic nose (E-nose) to explore the difference in scent information between PC and AC and distinguish them quickly and reliably. Meanwhile, the volatile components of these two herbs were detected by gas chromatography-mass spectrometry (GC-MS), and the results showed that 24 volatile components, such as 2,4-divert-butyl phenol, dodecane, and so on, can be used as chemical marker components to distinguish PC and AC [12]. The above research has contributed to identifying and analyzing the PC, AC, and LC to a certain extent, providing scientific evidence to avoid confusion and misuse. However, it is undeniable that there are still some limitations: (1) The above analysis often focused on one or two of the LC, PC, and AC and failed to place the three traditional Chinese medicines into a unified analysis system at the same time, which means the analysis results were relatively one-sided, and (2) due to the poor stability of volatile components, the volatile components in LC, PC, and AC would be lost in large quantities after being stored for some time. Therefore, the research of taking volatile components as the breakthrough point only applied to the analysis of fresh herbal medicines rather than the long-stored Chinese herbal medicines, making it difficult to be representative.

**Figure 1:**
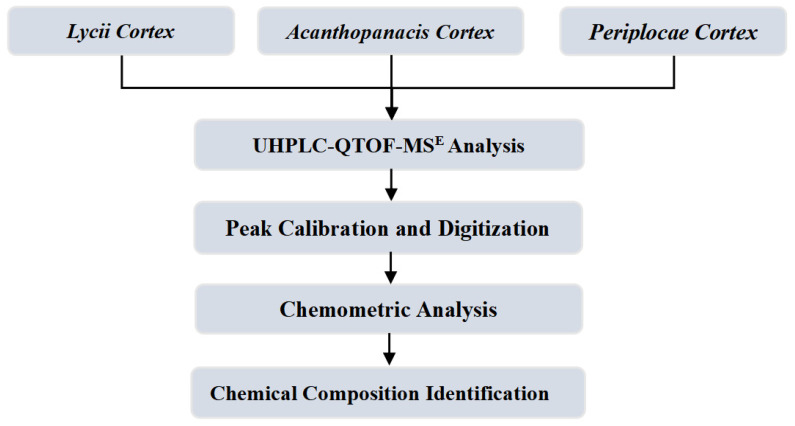
The overall research route.

Given the current analyses’ shortcomings, the non-volatile components were taken as the breakthrough point in this paper. LC, PC, and AC, stored for different periods, were selected as research objects to avoid the influence of unstable, volatile components and improve the representativeness of research and analysis. Furthermore, considering that UHPLC-QTOF-MS^E^ has the advantages of high separation efficiency, high resolution, and high sensitivity, it is an excellent method for component analysis, exploration of action mechanisms, and non-targeted identification of complex traditional Chinese medicines [13,14,15,16,17,18,19]. So, in this paper, the PC, LC, and AC samples were all analyzed by UHPLC-QTOF-MS^E^ and brought into a unified analysis system. The overall route of this research is shown in Figure 1. Firstly, the UHPLC-QTOF-MS^E^ technology was utilized to analyze PC, LC, and AC under the unified analysis conditions. Secondly, the Progenesis QI software (2.3 version) was used to perform peak position calibration and digitize the mass chromatography [20]. Then, the differential chemical components were explored combined with chemometric analysis in the SIMCA 14.1 software. Finally, according to the chemical standard substances, related literature, and databases, the differential chemical components of PC, LC, and AC were identified.

## 2. Results 

### 2.1. The Results of UHPLC-QTOF-MS^E^ Analysis

**Figure 2:**
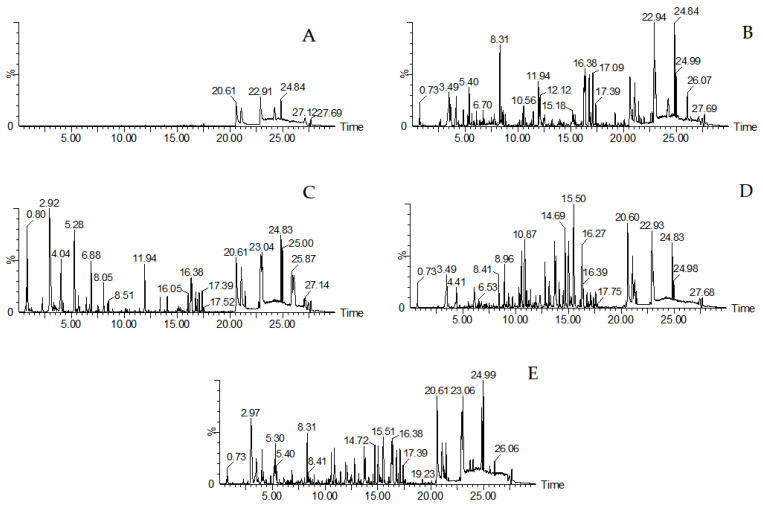
The base peak chromatogram of blank, PC, AC, LC, and the quality control sample ((**A**): blank; (**B**): PC, batch: 20210501; (**C**): LC, batch: 20130701; (**D**): AC, batch: 20170301; (**E**): QC sample).

In UHPLC-QTOF-MS^E^ analysis, we finally obtained the mass spectrometry information for chemical standard substances, which can be obtained from Figure S1, and the base-peak chromatogram of AC, PC, and LC is shown in Figure 2. The blank methanol had no obvious interference on the detection of the three herbs. AC, PC, and LC present different chromatograms, which suggests that the three herbs do have different chemical compositions. It laid a foundation for us to explore the differential chemical constituents of the three herbs. On the other hand, if our research were only based on the UHPLC-QTOF-MS^E^ analysis, it would be difficult to establish the characteristic relationship between chemical constituents and the three Chinese medicines, not to mention to explore different chemical compositions. Therefore, it was a good choice to combine the UHPLC-QTOF-MS^E^ data with chemometric analysis. 

### 2.2. The Results of Chemometric Analysis

**Figure 3:**
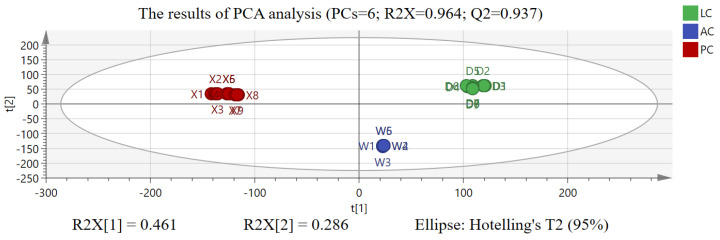
The results of score plots of Acanthopanacis Cortex (AC), Periplocae Cortex (PC), and Lycii Cortex (LC) in PCA analysis.

**Figure 4:**
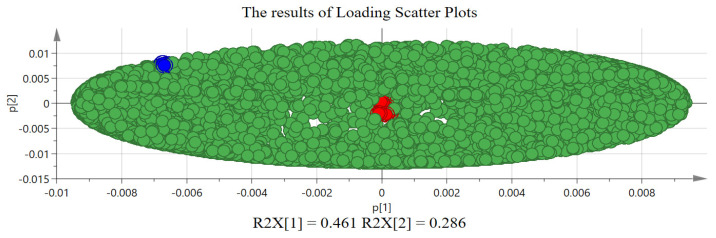
The results of loading scatter plots of Acanthopanacis Cortex (AC), Periplocae Cortex (PC), and Lycii Cortex (LC) in PCA analysis (Red dots: Variables that contribute less to PC1 and PC2; blue dots: Variables that contribute more to PC1 and PC2).

The quantized mass spectrometry data were normalized by unit variance scaling (UV); then, PCA analysis was carried out. The score plot and loading scatter plot of PCA analysis are shown in Figure 3 and Figure 4. LC, PC, and AC can be distinguished from each other effectively and clearly. At the same time, the cumulative interpretation rate (R2X [6]) of six principal components (PCs) is 0.964, and the cumulative prediction rate (Q2) is 0.937 [21,22]. On the other hand, the load and score plots are complementary illustrations of each other. The position of a sample in a given direction on the score plot is affected by a variable in the same direction on the load plot. In Figure 4, each point represents a variable whose position shows the importance of that variable in the principal component construction. Variables closer to the origin of the axes contribute less to the principal component, while variables further from the origin are more important [22]. For example, as shown in Figure 4, the red dots of 6.73 min_475.1785 *m*/*z*, 14.71 min_399.1827 *m*/*z*, 16.05 min_737.3172 *m*/*z*, 19.54 min_727.4019 *m*/*z*, etc., contribute less to PC1 and PC2, which is not conducive to the distinction between LC, PC, and AC. At the same time, the blue dots of 4.96 min_593.1910 *m*/*z,* 5.57 min_585.2092 *m*/*z*, 6.61 min_323.1269 *m*/*z*, 6.87_874.3739 *m*/*z*, 8.04 min_897.3897 *m*/*z,* 10.14 min_255.0869 *m*/*z*, etc., make great contributions to PC1 and PC2 and are negatively correlated with PC1 but positively correlated with PC2, which helps to distinguish AC, PC, and LC. Therefore, in differential chemical components analysis of PC, LC, and AC, we should pay more attention to the data points that are far from the origin of the coordinates. 

**Figure 5:**
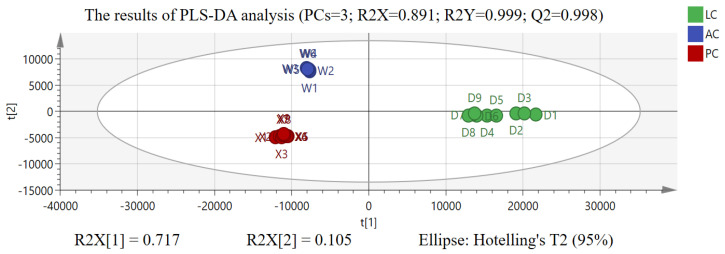
The results of score plots of *Acanthopanacis Cortex* (AC), *Periplocae Cortex* (PC), and *Lycii Cortex* (LC) in PLS-DA analysis.

**Figure 6:**
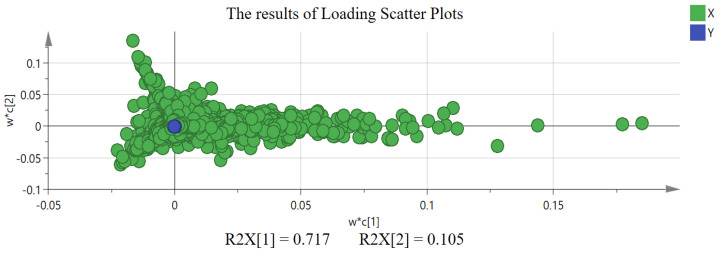
The results of loading scatter plots of *Acanthopanacis Cortex* (AC), *Periplocae Cortex* (PC), and *Lycii Cortex* (LC) in PLS-DA analysis.

Further, to explore the differential chemical components responsible for distinguishing AC, PC, and LC, the quantized mass spectrometry data were normalized by Pareto scaling (Par) to execute PLS-DA analysis. The score plot and loading scatter plot of PLS-DA analysis are shown in Figure 5 and Figure 6. LC, PC, and AC can also be clearly distinguished in supervised PLS-DA analysis, with the cumulative prediction rate (Q2) being 0.998. On the other hand, due to the PLS-DA model having the risk of overfitting, we further used 200 permutation tests and cross-validation analysis (CV-ANOVA) to assess whether the PLS-DA model was overfitting. The results of model validation show that the PLS-DA model is not overfitting with *p* = 3.85 × 10^−37^ < 0.01 [23,24,25,26]. 

Moreover, VIP was used to find component ions contributing to distinguishing LC, PC, and AC in the PLS-DA model. VIP > 1.0 is often considered a commonly used criterion for screening differential components [27,28]. 

**Figure 7:**
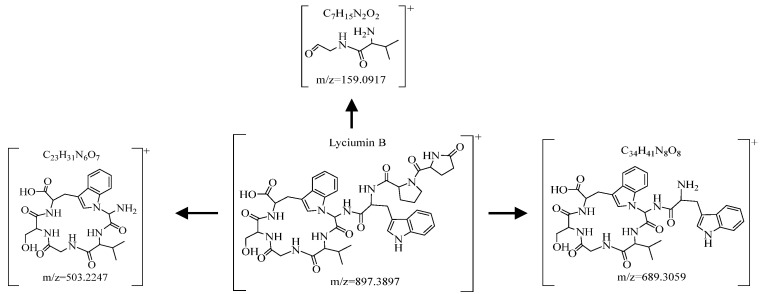
The fragmentation ion of Lyciumin B.

There were 2661 ions whose VIP values are more significant than 1.0. Based on the chemical standard substances, Waters UNIFI database, HMDB database, relevant literature reports, and our self-built database, we preliminarily identified the differential chemical constituents [29,30,31]. For example, the adduct ion of [M+H]^+^ of compound A was *m*/*z* = 897.3897, which showed that the molecular composition of compound A was C_44_H_52_N_10_O_11_. Moreover, five main fragments at *m*/*z* 879.3695 (C_44_H_51_N_10_O_10_), *m*/*z* 689.3059 (C_34_H_41_N_8_O_8_), *m*/*z* 503.2247 (C_23_H_31_N_6_O_7_), *m*/*z* 395.1719 (C_21_H_23_N_4_O_4_), and *m*/*z* 159.0917 (C_7_H_15_N_2_O_2_) were presented in the secondary mass spectrometry (MS2) to prove the identification analysis. After comparing with “Lyciumin B” of the chemical standard substances, Waters UNIFI database, the HMDB databases, and our self-built database, compound A was confirmed to be 11-(hydroxymethyl)-2-[[3-(1H-indol-3-yl)-2-[[1-(5-oxopyrrolidine-2-carbonyl)pyrrolidine-2-carbonyl]amino]propanoyl]amino]-3,6,9,12-tetraoxo-5-propan-2-yl-1,4,7,10,13-pentazatricyclo [14.6.1.017,22]tricosa-16(23),17,19,21-tetraene-14-carboxylic-acid (Lyciumin B) that was also included in the HMDB database, with the characteristic ion of *m*/*z* 879.3695 and data deviation being within 3.0 ppm [12,30]. As a cyclic peptide, Lyciumin B contains many easily broken peptide bonds (-CO-NH-), and the part ionic fragments after cleavage of Lyciumin B are shown in Figure 7.

**Figure 8:**
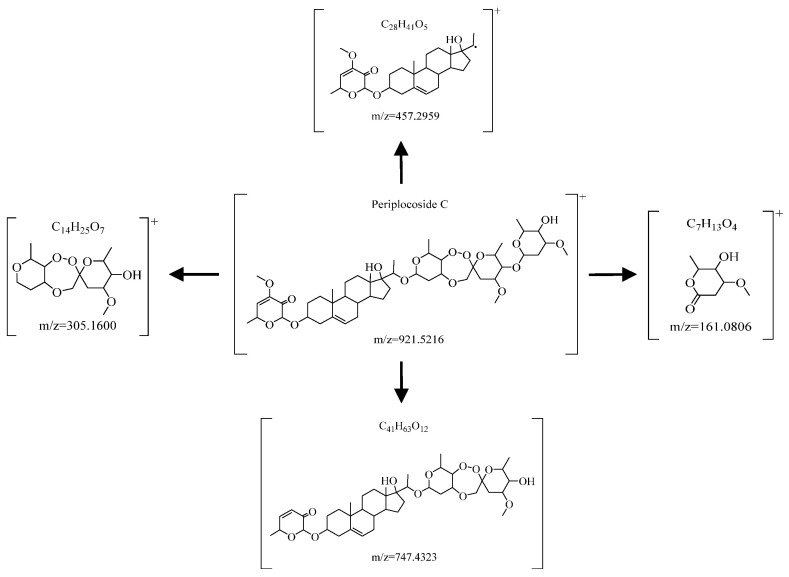
Part secondary cleavage ion fragments that assisted in identification of Periplocoside C.

The compound B appeared to be an adduct ion [M+H]^+^ at *m*/*z* = 921.5216, combined with its six secondary fragment ions *m*/*z* = 587.3598 (C_34_H_51_O_8_), *m*/*z* = 417.2136 (C_20_H_33_O_9_), *m*/*z* = 305.1600 (C_14_H_25_O_7_), *m*/*z* = 747.4313 (C_41_H_63_O_12_), *m*/*z* = 457.2959 (C_28_H_41_O_5_), and *m*/*z* = 161.0806 (C_7_H_13_O_4_). Through “Periplocoside C (*m*/*z* = 921.5276)” of the chemical standard substances and relevant reference literature comparison, we finally identified the compound C as Periplocoside C, and its accurate molecular formula is C_49_H_76_O_16_ with data deviation being within 2.0 ppm [32,33]. Since ether bonds are easily broken under ESI ionization, Periplocoside C containing multiple ether bonds (-C-O-C-) is usually cleaved into multiple secondary fragment ions. In addition, ether bonds are highly susceptible to free radical reactions. Based on the above analysis, the secondary cleavage ion fragments that assisted in the identification of Periplocoside C are shown in Figure 8.

**Figure 9:**
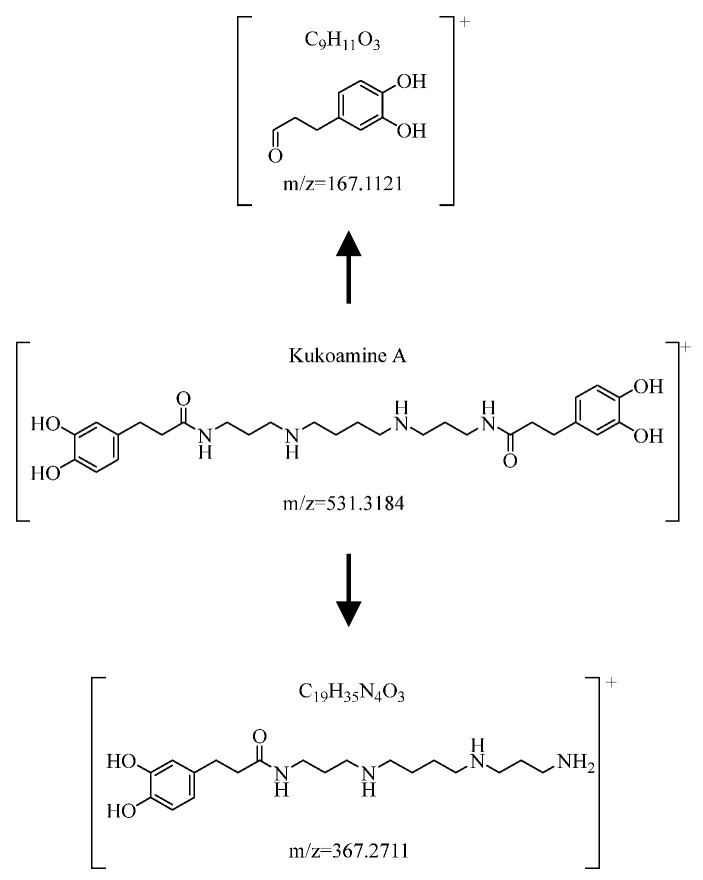
The fragmentation ions of Kukoamine A.

The adduct ion of [M+H]^+^ of compound C was *m*/*z* = 531.3184, which showed that the molecular composition of compound C was C_28_H_42_N_4_O_6_. Meanwhile, multiple fragment ions were presented in the MS2, such as *m*/*z* = 367.2711 (C_19_H_35_N_4_O_3_), *m*/*z* = 167.0721 (C_9_H_11_O_3_), *m*/*z* = 222.1112 (C_12_H_16_NO_3_), and *m*/*z* = 123.0441 (C_7_H_7_O_2_). After comparison with “Kukoamine A (*m*/*z* = 531.3195)” of the chemical reference substances and database retrieval combined with the attribution of ionic fragments, we identified compound C as Kukoamine A with data deviation within 3.0 ppm [30,34]. The detailed information of part fragment ions is shown in Figure 9.

The adduct ion of [M+Na]^+^ of compound D was *m*/*z* = 499.1244, which showed that the molecular composition of compound D was C_23_H_24_O_11_ with data deviation within 5.6 ppm. At the same time, three fragment ions *m*/*z* = 315.0693 (C_17_H_15_O_6_), *m*/*z* = 171.1120 (C_8_H_11_O_4_), *m*/*z* = 163.0667 (C_6_H_11_O_5_), and *m*/*z* = 145.0286 (C_6_H_9_O_4_) were obviously present in the high energy channel to assist in the verification of compound D [35]. Based on the fragment cleavage pattern and UNIFI database comparison, we identified compound D as likely to be 5-Hydroxy-6,7-dimethoxyflavone-4′-*O*-beta-D-glucopyranoside.

The compound E appeared to be an adduct ion [M+H]^+^ at *m*/*z* = 153.0555 with data deviation within 2.0 ppm, and there were three cleavage ion fragments *m*/*z* = 135.0420 (C_8_H_7_O_2_), *m*/*z* = 125.0600 (C_7_H_9_O_2_), and 121.0285 (C_7_H_5_O_2_) in the high-energy channel. Based on the comparison with “2-Hydroxy-4-methoxybenzaldehyde (*m*/*z* = 153.0563)” of the chemical reference substances and HMDB database, we finally identified compound E as 2-Hydroxy-4-methoxybenzaldehyde [30,32,34].

As shown in Table 1, we preliminary identified 17 differential chemical constituents whose VIP > 1.0 in AC, PC, and LC. More information is detailed in Table S2.

Furthermore, we used a nonparametric rank sum test (data non-normal distribution) to verify whether there is a significant difference for the 17 differential chemical components in LC, AC, and PC. As shown in Table 2, the probability values of the 17 compounds were all < 0.01, which showed that the identified 17 chemical constituents had substantial differences between PC, AC, and LC. For example, Kukoamine A (C_28_H_42_N_4_O_6_, *m*/*z* = 531.3184) and Lyciumin A (C_42_H_51_N_9_O_12_, *m*/*z* = 874.3735), as well as Lyciumin B (C_42_H_51_N_9_O_12_, *m*/*z* = 897.3895), in LC had a higher ionic strength compared to PC and AC (basically at the baseline level), while the Periploside C (C_44_H_52_N_10_O_11_, *m*/*z* = 921.5216) and Periplocoside (C_36_H_56_O_13_, *m*/*z* = 719.3613) in PC had a higher ionic strength. In conclusion, we identified 17 differential compounds that were expected to be potential chemical markers for differentiating the PC, AC, and LC.

## 3. Discussion

### 3.1. Optimization of Analysis Conditions

Before the formal experiment, we optimized the analysis conditions through the pre-trial. As far as sample pretreatment is concerned, we found that the extraction effect of methanol was better than that of 50% methanol-water and ethyl acetate. As for mass spectrometry, the mass spectrometry information in the positive ion mode was more abundant than in the negative ion mode. The MS^E^ mode was used to collect the mass spectrometry data, in which collision energy will change from low to high energy circularly, thereby ensuring the simultaneous collection of precursor ions and fragment ions and facilitating the subsequent database retrieval and literature comparison. In the investigation of collision energy, it was distinct that the mass spectrometry had the most abundant data information with the collision energy being 10–40 V, rather than 10–60 V or 10–80 V.

### 3.2. Discussion on Chemometric Analysis

Generally speaking, PCA and PLS-DA are the most commonly used analytical methods to explore the differential chemical components in chemometric analysis [22,37]. Drawing on their successful application in Chinese medicine, we also used PCA and PLS-DA to explore the different chemical compositions of LC, AC, and PC. Moreover, before PCA and PLS-DA analysis, the data need to be normalized; unit variance scaling (UV) and Pareto scaling (Par) are the common normalization methods for PCA and PLS-DA, respectively [12,22,37,38]. In addition, if the PLS-DA model is overfitting, the analysis result will be unreliable. Therefore, permission tests and cross-validation were used to verify the reliability of the PLS-DA model in this paper. In addition, the VIP in the PLS-DA model can help us to quickly lock the most important ions for distinguishing the three herbs from a large amount of complex ion information.

### 3.3. Discussion on Sample Representativeness

The LC, PC, and AC samples, known as reference herbs, come from the National Institutes for Food and Drug Control. The herbs are high-quality traditional Chinese medicines of different origins, such as Sichuan, Shanxi, and Zhejiang, and they were used as control reference in the process of traditional Chinese medicine testing. Its collection must be strictly standardized for origin and harvesting, and the species and origin are entirely accurate and verified by experts, aligning with *Chinese Pharmacopoeia*’s quality requirements. Therefore, the samples of different origins and different collection years are representative. Moreover, the analysis found that the mass spectrometry information of the same herbs of different origins and storage time varied, especially those preserved for a longer time with the least mass spectrometry information due to the loss of volatile components [12]. Therefore, based on the representative samples from various sources and with different storage times, we could control the chemical composition differences to the greatest extent and realize accurate chemometric analysis. In addition, all samples were pulverized in the year of collection and were kept in a standard library of control herbs before formal analysis. In summary, the samples are representative to some degree. However, it is undeniable that the number of samples used in this study is indeed small. Still, the samples involve different years and origins, and it takes time to collect them, so it will be necessary to further increase the sample size for analysis and validation in subsequent studies.

### 3.4. Discussion on Analysis Results

Traditional Chinese medicine is a multi-component system containing thousands of chemical components, with a large amount of chemical information that can reflect the species characteristics of the sample. After performing a chemometric analysis on AC, PC, and LC, we could characterize the chemical composition and species relationship. In this paper, we have identified 17 potential differential chemical components, including unique chemical components and shared chemical components whose content has significant differences. For example, Lyciumin B is the unique component of LC, and Periploside H2 is the unique component of PC. The content of these chemical components will vary with individual differences, but it remains the key chemical marker for identifying and distinguishing the three Chinese medicines. The shared chemical components were present in AC, LC, and PC, but the compositional content, such as N-Feruloyltyramine, varied greatly. Further, considering the representativeness of samples from the National Institutes for Food and Drug Control collected from different producing areas, the 17 potential chemical markers currently identified are representative enough to realize the identification and analysis of AC, LC, and PC. In summary, we can use the 17 potential chemical markers to realize the market supervision and quality control of AC, LC, and PC, and perhaps we can take the following measures: (1) For proprietary chemical components, Lyciumin B and Periploside H2 are the proprietary chemical components of LC and PC, respectively. So we can stipulate that Lyciumin B should not be detected in PC and AC and Periploside H2 should not be detected in LC and AC, etc. (2) For shared chemical components, such as 2-Hydroxy-4-methoxybenzaldehyde, Periplocoside K, Periplocoside C, 3,5-Di-O-caffeoylquinic acid, etc., we can enhance the quality control of LC, PC, and AC by setting content limits to these chemical components. (3) Perhaps multiple ingredients including proprietary and shared chemical components can be used as an “ingredients combination”, and when all of them can be detected, it is deemed that the herb has been detected.

### 3.5. Research Advantage, Limitation, and Prospect

In this paper, UHPLC-QTOF-MS^E^ combined with chemometric analysis was used to explore the different chemical compositions. Further, 17 differential chemical components were identified according to the chemical standard substances, related literature, and databases. These chemical components may be potential quality control markers to distinguish LC, PC, and AC. It is beneficial to strengthen the quality control and market supervision of LC, PC, and AC. Therefore, in terms of practicality, UHPLC-QTOF-MS^E^ combined with chemometric analysis helps explore the differential chemical components of similar herbs and realize the identification of similar herbs based on differential chemical markers. However, this method has a high cost and low analysis speed. Samples need to be extracted and analyzed by personnel with the necessary professional knowledge. Moreover, as mentioned before, although the samples are representative to some extent, the number of samples is small; so, it is necessary to increase the sample size for analysis and verification in the future. In addition, from the point of view of compositional identification, we are still determining what most compounds are. It is well known that the composition of traditional Chinese medicine is very complex, containing thousands of compounds, and the identification of compounds is time-consuming, laborious, and may not be correct. Therefore, rational utilization of information on unknown components in traditional Chinese medicines to facilitate quality control of traditional Chinese medicines may be a direction for future research and development.

## 4. Materials and Methods

### 4.1. Experimental Materials

A total of 24 standard samples belonging to the three species, including AC (6 cortex samples), LC (9 cortex samples), and PC (9 cortex samples), were collected from the National Institutes for Food and Drug Control. All samples were identified by the laboratory and met the requirements of the 2020 edition of *Chinese Pharmacopoeia*; the detailed information, such as number, year, name, and so on, about standard samples is shown in Table S1. In addition, all the samples were pulverized in the year of collection and stored in a cool and dry place of a standard library of control herbs. The chemical reference standards of 3,5-Di-O-caffeoylquinic acid, Lyciumin A, Lyciumin B, Periplocoside, Periplocoside C, Periplocoside B, Periplocoside, Kukoamine A, 2-Hydroxy-4-methoxybenzaldehyde, 7-Methoxycoumain, N-caffeoyltyramine, etc. were purchased from Sunshine Trading Co., Ltd., Hong Kong, China.

### 4.2. Reagent Materials

Mass spectrometry-grade methanol (lot: ED341-CN) was purchased from Honeywell Trading Co., Ltd. of Shanghai, China. Mass spectrometry-grade acetonitrile (lot: 222372) was purchased from Thermo Fisher Scientific Technology Co., Ltd. of Shanghai, China. Mass spectrometry-grade formic acid (L1670) was purchased from Honeywell Trading Co., Ltd. of Shanghai, China. Ultrapure water (GB 19298) was purchased from Watsons Food and Beverage Co., Ltd. of Guangzhou, China.

### 4.3. Sample Pretreatment and UHPLC-QTOF-MS^E^ Analysis

The 10.00 mg of each standard was weighed precisely, and the solution was diluted to 200 mL; then, 1.00 mL was pipetted and diluted to 200 mL again to make the standard solution with a concentration of 250 ng/mL for identification of chemical composition. The specific procedure for herbal samples pretreatment is as follows: firstly, accurately weigh 1.00 g of herbal material dried powder of AC, PC, and LC, and place in 50 mL tapered bottle with a plug respectively; then, accurately add 25.00 mL of mass-spectrum methanol with a pipette into tapered bottle to perform ultrasound for 30 min (power: 500 W, frequency: 40 kHz); finally, take out and cool to room temperature and filter with 0.22 μm organic filter membrane to obtain samples to be analyzed. The samples were stored at 4 °C in the refrigerator before UHPLC-QTOF-MS^E^ analysis. In addition, the quality control sample was a mixed AC, PC, and LC solution.

The UHPLC-QTOF-MS^E^ analysis was performed using liquid chromatography tandem time-of-flight mass spectrometry on Waters Xevo G2-XS QTof (Waters, United States of America, USA). Chromatographic separations were conducted on Waters Acquity UHPLC BEH-C_18_ (2.1 mm × 100 mm, 1.7 μm) chromatographic column (lot: 186002352 Waters, United States of America, USA). For the analysis of AC, PC, and LC samples, the column temperature was programmed at 35 ℃. The mobile phases were 0.1% formic acid in water (A phase)-acetonitrile (B phase), and the gradient elution conditions were as follows: 0–23 min, 5–95% B; 23–26 min, 95% B; 26–26.01 min, 95–5% B; and 26.01–30 min, 5% B. The injection volume was 2.0 μL. On the other hand, the ESI-positive ionization mode was used for detection and analysis in this study. The MS^E^ data acquisition method was used in which the data acquisition rate was set to 0.2 s; The scanning range of m/z was 100–1500; the collision gas was high purity Argon, and the real-time mass axis calibration solution (lock mass) was Leucine Enkephalin (LE) whose concentration was 300 ng/mL. In addition, capillary: 3.0 kV; sampling cone: 40 V; source offset: 80 V; desolvation temperature: 450 °C; desolvation gas: 900 L/h, collision energy:10–40 V; and source temperature: 120 °C. The mass axis and lock mass were calibrated before sample analysis.

### 4.4. Data Processing and Analysis

The mass spectrometry information of AC, PC, and LC was processed by Progenesis QI software (version 2.3) with the parameters as follows: type of machine: high-resolution mass spectrometer; ionization polarity: positive; retention time: 1.00~26.00 min; peak picking limits: automatic; and Rt window: 0.1 min. We obtained the quantized data, including retention time (Rt), mass-to-charge ratio (*m*/*z*), and ionic strength (I). SIMCA P 14.1 (Umetrics, Umeå, Sweden) was used for data analysis, in which PCA and PLS-DA were adopted to explore the differential chemical composition ions based on VIP [22,37,38,39,40]. Further, the differential chemical components of LC, AC, and PC were identified according to the chemical standard substances, related literature, and databases and verified based on mathematical statistical analysis.

## 5. Conclusions

In this paper, UHPLC-QTOF-MS^E^ and chemometric analysis were successfully applied to explore the differential chemical components of PC, AC, and LC. Moreover, 17 differential chemical constituents were identified according to the chemical standard substances, related literature, and databases and verified based on non-parametric test. The proprietary ingredients, Lycium B (LC) and Periploside H2 (PC), are beneficial in strengthening the quality control of AC, PC, and LC. In addition, limits on the content of shared components can also be set to enhance the quality control of LC, PC, and AC. This study is beneficial for strengthening the quality control of these three Chinese medicines.

## Figures and Tables

**Table 1:** The detailed information of the 17 chemical components.

Composition	*m*/*z*	Fragment Ions	Adduct Ion	Molecular Formula
3,5-Di-O-caffeoylquinic acid	517.1357	499.1244\355.1080\145.0284\135.0427	[M+H]^+^	C_25_H_24_O_12_
Lyciumin A	874.3739	856.3706\666.2883\503.2260\486.2028 372.1536\181.0995	[M+H]^+^	C_42_H_51_N_9_O_12_
Lyciumin B	897.3897	879.3695\851.3809\689.3059\643.2662\503.2247\395.1719\181.1010\159.0917	[M+H]^+^	C_44_H_52_N_10_O_11_
Periploside H2	1165.6003	819.4387\703.4024\657.3876 485.1868\323.1347\315.1425\203.0941\171.0657	[M+H]^+^	C_56_H_92_O_25_
Periplocoside C	921.5216	161.0806\203.0914\305.1600\417.2136\587.3598\747.4313	[M+H]^+^	C_49_H_76_O_16_
Periplocoside B	1065.5992	1035.5977\921.5239\747.4311\551.3381\439.2844\417.2163	[M+H]^+^	C_56_H_88_O_19_
Periplocoside	719.3613	665.3542\535.3222\391.2525\373.1476\355.2287\337.2187\275.1131	[M+Na]^+^	C_36_H_56_O_13_
3-*O*-(β-D-glucopyranose (1→2)-β-D-glucopyranose)-16α-ethoxy -oleanolic acid-28-*O*-β-D-glucopyranoside	1009.5346	807.4570\687.4120\371.1729\337.2332\291.1958\162.1353	[M+Na]^+^	C_50_H_82_O_19_
Kukoamine A	531.3184	367.2711\293.1868\251.1363\222.1112\167.0721\165.0528\123.0441	[M+H]^+^	C_28_H_42_N_4_O_6_
5-Hydroxy-6,7-dimethoxyflavone-4′-*O*-beta-D-glucopyranoside	499.1244	315.0693\171.1120\163.0667\145.0286	[M+Na]^+^	C_23_H_24_O_11_
2-Hydroxy-4-methoxybenzaldehyde	153.0555	135.0420\125.0600\121.0285	[M+H]^+^	C_8_H_8_O_3_
Periplocoside K	825.4255	629.2805\485.1865\325.1138\323.1341\203.0940	[M+Na]^+^	C_40_H_66_O_16_
N-FeruloyltyraMine	314.1392	177.0548\134.0356	[M+H]^+^	C_18_H_19_NO_4_
N-caffeoyltyramine	300.1238	121.0640	[M+H]^+^	C_17_H_17_NO_4_
3-*O*-acetyl-caffeic acid	223.0606	123.0443\134.0367	[M+H]^+^	C_11_H_10_O_5_
1-Monopalmitin	353.2667	313.2765\239.2404	[M+Na]^+^	C_19_H_38_O_4_
7-methoxycoumain [36]	177.0552	162.0364\149.0584\133.0698\118.0383	[M+H]^+^	C_10_H_8_O_3_

**Table 2:** The nonparametric test results of the 17 chemical components.

Compounds	Class Median			Kruskal-Wallis-H Value	*p*
	LC (n = 9)	AC (n = 6)	PC (n = 9)		
3,5-Di-O-caffeoylquinic acid	5830.110 (2778.9, 9303.2)	184,194.500 (181,343.5, 194,189.5)	12,993.500 (11,643.9, 16396.9)	20.25	0.000 **
Lyciumin A	1,600,530.000 (355,550.0, 2,149,495.0)	494.760 (487.2, 499.8)	1889.880 (1871.3, 1922.7)	20.25	0.000 **
Lyciumin B	34,775.800 (26,950.8, 55,647.2)	0.000 (0.0, 0.0)	0.000 (0.0, 0.0)	21.41	0.000 **
Periploside H2	26.202 (0.0, 24,765.5)	82.655 (71.5, 107.0)	292,979.000 (281,991.0, 353,838.5)	16.72	0.000 **
Periplocoside C	11,552.900 (10,465.3, 31,519.0)	26,186.300 (25,263.7, 26,754.5)	179,990.000 (140,397.0, 182,907.0)	16.65	0.000 **
Periplocoside B	8872.360 (8011.5, 44,087.2)	23,943.500 (23,182.0, 24,265.3)	152,935.000 (133,812.0, 212,033.0)	16.65	0.000 **
Periplocoside	262.189 (167.4, 11,596.4)	2095.605 (1985.1, 2163.4)	28,947.300 (27,909.1, 39,741.9)	16.65	0.000 **
3-O-(β-D-glucopyranose (1→2)-β-D-glucopyranose) -16α-ethoxy-oleanolic acid -28-O-β-D-glucopyranoside	0.000 (0.0, 5724.1)	3487.590 (3211.4, 3559.4)	31,907.300 (23,078.8, 45,327.4)	16.91	0.000 **
Kukoamine A	6,393,320.000 (4,404,335.0, 6,703,815.0)	0.000 (0.0, 0.0)	1055.530 (292.7, 1394.1)	20.56	0.000 **
5-Hydroxy-6,7-dimethoxyflavone-4′-O-beta-D-glucopyranoside	3283.940 (755.7, 5009.4)	341,882.000 (334,246.8, 366,415.5)	34,851.100 (22,416.1, 37,870.1)	20.25	0.000 **
2-Hydroxy-4-methoxybenzaldehyde	217.577 (95.0, 1380.7)	3061.145 (3021.6, 3138.2)	50,979.300 (20,609.9, 57,093.0)	20.25	0.000 **
Periplocoside K	3144.210 (224.8, 8289.9)	2119.750 (2048.7, 2207.6)	13,786.500 (12,795.0, 14,256.9)	16.25	0.000 **
N-FeruloyltyraMine	237,802.000 (146,926.5, 781,073.0)	1271.425 (1259.4, 1344.9)	1012.490 (786.3, 1113.7)	18.89	0.000 **
N-caffeoyltyramine	246,638.000 (85,521.4, 348,217.0)	530.405 (501.3, 557.8)	1011.030 (581.5, 1052.8)	19.40	0.000 **
3-O-acetyl-caffeic acid	18,414.500 (8236.1, 25,494.3)	600.173 (542.5, 667.1)	25,812.000 (18,788.3, 37,798.0)	15.01	0.001 **
1-Monopalmitin	377,254.000 (331,131.0, 602,292.0)	63,102.600 (57,234.3, 67,633.6)	36,612.100 (29,466.7, 42,631.4)	20.25	0.000 **
7-methoxycoumain	1629.570 (1294.6, 3267.7)	602.553 (579.9, 627.4)	20,946.200 (13,503.5, 23,814.5)	20.25	0.000 **

** *p* < 0.01.

## Data Availability

The data information can be obtained from Supplementary Materials.

## Supplementary Materials

The following supporting information can be downloaded at: https://www.mdpi.com/article/10.3390/molecules29163807/s1, Table S1: the detailed information of *Acanthopanacis Cortex*, *Lycii Cortex*, and *Periplocae Cortex* samples; Table S2: specific information of compounds and fragment ions; Figure S1: the mass spectrogram of chemical reference standards used for identifying compounds.
