# Supplementary material for: Differential Chemical Components Analysis of Periplocae Cortex, Lycii Cortex, and Acanthopanacis Cortex Based on Mass Spectrometry Data and Chemometrics
Source: Molecules. 2024 Aug 11;29(16):3807. doi: 10.3390/molecules29163807 (PMC11357377; doi:10.3390/molecules29163807)
Supplement: Supplementary file 1 [file molecules-29-03807-s001.zip › Table S1.pdf]

Table S1. The detailed information of Acanthopanacis Cortex, Lycii Cortex and Periplocae Cortex samples.

Chinese Medicine Name	Batch number	Sample Source	Years	Place of origin
Acanthopanacis Cortex	AC01	National Institutes for Food and Drug Control	2008	Hubei, China
Acanthopanacis Cortex	AC02	National Institutes for Food and Drug Control	2008	Hubei, China
Acanthopanacis Cortex	AC03	National Institutes for Food and Drug Control	2008	Hubei, China
Acanthopanacis Cortex	AC04	National Institutes for Food and Drug Control	2017	Henan, China
Acanthopanacis Cortex	AC05	National Institutes for Food and Drug Control	2017	Henan, China
Acanthopanacis Cortex	AC06	National Institutes for Food and Drug Control	2017	Henan, China
Lycii Cortex	LC01	National Institutes for Food and Drug Control	2012	Shanxi, China
Lycii Cortex	LC02	National Institutes for Food and Drug Control	2012	Shanxi, China
Lycii Cortex	LC03	National Institutes for Food and Drug Control	2012	Shanxi, China
Lycii Cortex	LC04	National Institutes for Food and Drug Control	2013	Zhejiang, China
Lycii Cortex	LC05	National Institutes for Food and Drug Control	2013	Zhejiang, China
Lycii Cortex	LC06	National Institutes for Food and Drug Control	2013	Zhejiang, China
Lycii Cortex	LC07	National Institutes for Food and Drug Control	2017	Jiangsu, China
Lycii Cortex	LC08	National Institutes for Food and Drug Control	2017	Jiangsu, China
Lycii Cortex	LC09	National Institutes for Food and Drug Control	2017	Jiangsu, China
Periplocae Cortex	PC01	National Institutes for Food and Drug Control	2005	Sichuan, China
Periplocae Cortex	PC02	National Institutes for Food and Drug Control	2005	Sichuan, China
Periplocae Cortex	PC03	National Institutes for Food and Drug Control	2005	Sichuan, China
Periplocae Cortex	PC04	National Institutes for Food and Drug Control	2014	Shandong, China
Periplocae Cortex	PC05	National Institutes for Food and Drug Control	2014	Shandong, China
Periplocae Cortex	PC06	National Institutes for Food and Drug Control	2014	Shandong, China
Periplocae Cortex	PC07	National Institutes for Food and Drug Control	2021	Hebei, China
Periplocae Cortex	PC08	National Institutes for Food and Drug Control	2021	Hebei, China
Periplocae Cortex	PC09	National Institutes for Food and Drug Control	2021	Hebei, China
