# Supplementary material for: Differential Chemical Components Analysis of Periplocae Cortex, Lycii Cortex, and Acanthopanacis Cortex Based on Mass Spectrometry Data and Chemometrics
Source: Molecules. 2024 Aug 11;29(16):3807. doi: 10.3390/molecules29163807 (PMC11357377; doi:10.3390/molecules29163807)
Supplement: Supplementary file 1 [file molecules-29-03807-s001.zip › Table S2.pdf]

Table S2. specific information of compounds and fragment ions.

Serial Number	Compound Name	Measured ion(H or Na)	Fragment ions	Addition ion	Structural Formula	Theoretical molecular weight	Deviation(ppm)	VIP value
1	3,5-Di-O-caffeoylquinic acid	517.14	135.0427(C8H7O2)\145.0284(C9H5O2)\355.1080(C16H19O9)\499.1244(C25H23O11)	H	C25H24O12	517.1357	2.1	5.6
2	Lyciumin A	874.37	181.0995(C9H13N2O2)\372.1536(C19H22N3O5)\486.2028(C23H28N5O7)\503.2260(C23H31N6O7)\666.2883(C32H40N7O9)\856.3706(C42H50N9O11)	H	C42H51N9O12	874.3739	0.5	6.9
3	Lyciumin B	897.39	879.3924（C44H51N10O10）\851.3809(C43H51N10O9)\689.3060(C34H41N8O8)\643.2662(C32H35N8O7)\503.2247(C23H31N6O7)\395.1719(C21H23N4O4)\181.1010(C9H13N2O2)\159.0917(C7H15N2O2)	H	C44H52N10O11	897.3897	0.2	7.3
4	periploside H2	1165.60	819.4387(C40H67O17)\703.4024(C39H59O11)\657.3876(C34H57O12)\485.1868(C19H33O14)\323.1347(C13H23O9)\315.1425(C15H23O7)\203.0941(C9H15O5)\171.0657(C8H11O4)	H	C56H92O25	1165.6003	-0.3	6.7
5	Periplocoside C	921.52	161.0806(C7H13O4)\203.0914(C9H15O5)\305.1600(C14H25O7)\417.2136(C20H33O9)\439.2844(C28H39O4)\457.2959(C28H41O5)\587.3598(C34H51O8)\747.4313(C41H63O12)	H	C49H76O16	921.5216	0.4	4.4
6	Periplocoside B	1065.60	1035.5977(C55H87O18)\921.5239(C49H77O16)\747.4311(C41H63O12)\551.3381(C34H47O6)\439.2844(C28H39O4)\417.2163(C20H33O9)	H	C56H88O19	1065.5992	-0.6	4.4
7	Periplocoside	719.36	665.3542(C35H53O12)\535.3222(C30H47O8)\391.2525(C23H35O5)\373.1476(C23H33O4)\355.2287(C23H31O3)\337.2187(C23H29O2)\275.1131(C12H19O7)	Na	C36H56O13	719.3613	-0.8	2.1
8	3-O-(β-D- glucopyranose	1009.53	807.4570(C43H67O14)\687.4120(C39H59O10)\371.1729(C18H2	Na	C50H82O19	1009.5346	-0.2	2.0

	(1-2)-β-D- glucopyranose) -16α- ethoxy-oleanolic acid -28-O-β-D- glucopyranoside		7O8)\337.2332(C20H33O4)\291.1958(C18H27O3)\255.2105（C19H27）\162.1353(C6H10O5)					
9	Kukoamine A	531.32	367.2716(C19H35N4O3)\293.1868(C16H25N2O3)\251.1363(C13H19N2O3)\222.1112(C12H16NO3)\167.1121(C9H11O3)\165.0528(C9H9O3)\123.0444(C7H7O2)	H	C28H42N4O6	531.3184	0.2	14.8
10	5-Hydroxy-6,7-dimethoxy flavone-4'-O-beta-D-gluco pyranoside	499.12	315.0693(C17H15O6)\171.1120(C8H11O4)\163.0667(C6H11O5)\145.0286(C6H9O4)	Na	C23H24O11	499.1244	5.6	7.5
11	2-Hydroxy-4-methoxyben zaldehyde	153.06	135.0420(C8H7O2)\125.0600(C7H9O2)\121.0285(C7H5O2)\110.0370(C6H6O2)	H	C8H8O3	153.0555	2.0	2.2
12	Periplocoside K	825.43	629.2805(C30H45O14)\485.1865(C19H33O14)\325.1138(C12H21O10)\323.1341(C13H23O9)\203.0940(C9H15O5)	Na	C40H66O16	825.4255	0.7	1.3
13	N-FeruloyltyraMine	314.14	177.0548(C10H9O3)\134.0356(C8H6O2)	H	C18H19NO4	314.1392	0.0	3.4
14	N-caffeoyltyramine	300.12	121.0640(C8H9O)	H	C17H17NO4	300.1238	0.7	2.9
15	3-O- acetyl-caffeic acid	223.06	123.0443(C7H7O2)\134.0367(C8H6O2)	H	C11H10O5	223.0606	0.0	2.0
16	1-Monopalmitin	353.27	313.2765(C19H37O3)\239.2404(C16H31O)	Na	C19H38O4	353.2667	-0.3	3.7
17	7-Methoxycoumain	177.06	162.0364(C9H6O3)\149.0584(C8H5O3)\133.0698(C8H5O2)\118.0383(C7H2O2)	H	C10H8O3	177.0552	0.0	1.6
