# Supplementary figures and images for: Differential Chemical Components Analysis of Periplocae Cortex, Lycii Cortex, and Acanthopanacis Cortex Based on Mass Spectrometry Data and Chemometrics
Source: Molecules. 2024 Aug 11;29(16):3807. doi: 10.3390/molecules29163807 (PMC11357377; doi:10.3390/molecules29163807)

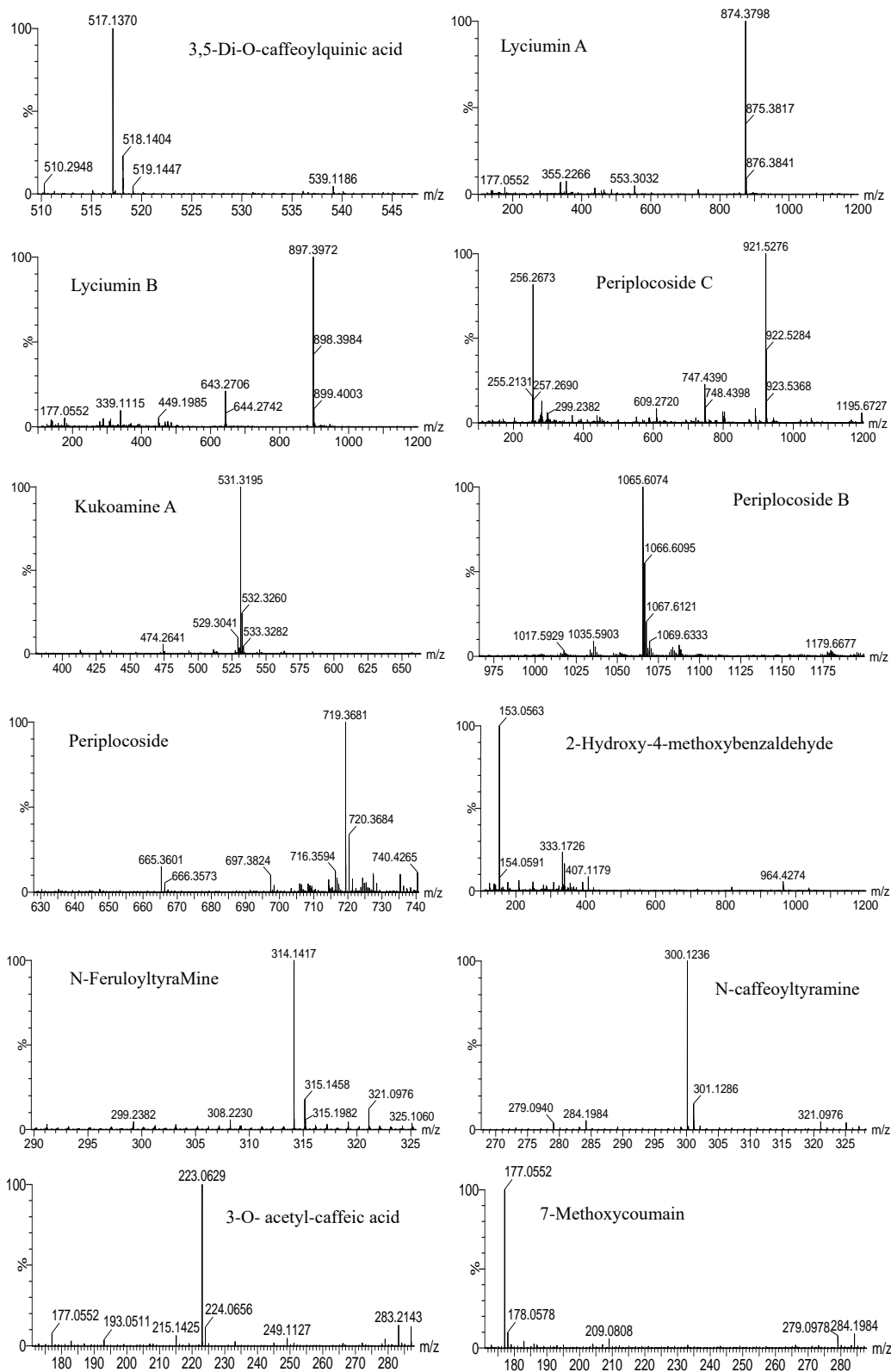

Figure S1. The mass spectrometry of chemical references used for identifying compounds.

Supplement: Supplementary file 1 [file molecules-29-03807-s001.zip › Figure S1.pdf]
